# Insulin-Like Growth Factor 1 Attenuates the Pro-Inflammatory Phenotype of Neutrophils in Myocardial Infarction

**DOI:** 10.3389/fimmu.2022.908023

**Published:** 2022-07-15

**Authors:** Rianne Nederlof, Sophia Reidel, André Spychala, Stefanie Gödecke, André Heinen, Tobias Lautwein, Patrick Petzsch, Karl Köhrer, Axel Gödecke

**Affiliations:** ^1^ Institut für Herz- und Kreislaufphysiologie, Medizinische Fakultät und Universitätsklinikum Düsseldorf, Heinrich-Heine-Universität Düsseldorf, Düsseldorf, Germany; ^2^ Biologisch-Medizinisches Forschungszentrum (BMFZ), Genomics and Transcriptomics Labor, Heinrich-Heine-Universität Düsseldorf, Düsseldorf, Germany; ^3^ Cardiovascular Research Institute Düsseldorf (CARID), Medizinische Fakultät und Universitätsklinikum Düsseldorf, Heinrich-Heine-Universität Düsseldorf, Düsseldorf, Germany

**Keywords:** insulin-like growth factor 1, myocardial infarction, neutrophil, signaling, inflammation

## Abstract

Acute myocardial infarction (MI) induces an extensive sterile inflammation, which is dominated in the early phase by invading neutrophils and monocytes/macrophages. The inflammatory response after MI critically affects infarct healing and cardiac remodeling. Therefore, modulation of cardiac inflammation may improve outcome post MI. Insulin-like growth factor 1 (IGF1) treatment reduces infarct size and improves cardiac function after MI *via* IGF1 receptor mediated signaling in myeloid cells. Our study aimed to investigate the effect of IGF1 on neutrophil phenotype both *in vitro* and *in vivo* after MI. We show that IGF1 induces an anti-inflammatory phenotype in bone marrow derived neutrophils. On the molecular and functional level IGF1 treated neutrophils were indistinguishable from those induced by IL4. Surprisingly, insulin, even though it is highly similar to IGF1 did not create anti-inflammatory neutrophils. Notably, the IGF1 effect was independent of the canonical Ras/Raf/ERK or PI3K/AKT pathway, but depended on activation of the JAK2/STAT6 pathway, which was not activated by insulin treatment. Single cell sequencing analysis 3 days after MI also showed that 3 day IGF1 treatment caused a downregulation of pro-inflammatory genes and upstream regulators in most neutrophil and many macrophage cell clusters whereas anti-inflammatory genes and upstream regulators were upregulated. Thus, IGF1 acts like an anti-inflammatory cytokine on myeloid cells *in vitro* and attenuates the pro-inflammatory phenotype of neutrophils and macrophages *in vivo* after MI. IGF1 treatment might therefore represent an effective immune modulatory therapy to improve the outcome after MI.

## Introduction

The immune system plays an important role in tissue damage and cardiac repair after acute myocardial infarction (AMI). The early phase after the onset of cardiac ischaemia is dominated by the innate immune system. Neutrophils are among the first cells that are recruited to the infarcted area, and reach their peak one day after MI (1). Neutrophils clear the infarcted area of cell debris, but also increase injury by producing pro-inflammatory cytokines, chemokines and high amounts of reactive oxygen species (ROS) (respiratory burst) (2). In addition, they attract pro-inflammatory Ly6C^hi^ monocytes, which may differentiate to pro-inflammatory macrophages, and thereby contribute to the generation of a pro-inflammatory environment (3).

Since neutrophils enhance inflammation, they are generally thought to have a negative effect on cardiac remodeling. However, different mechanisms have been published that show that neutrophils can also positively affect remodeling of the heart after MI. One of the mechanisms is that apoptotic neutrophils undergo phagocytosis by macrophages, which promotes them to release anti-inflammatory and reparative cytokines (4). Furthermore, neutrophil depletion reduces the polarization of macrophages to an anti-inflammatory M2c phenotype after MI and leads to increased fibrosis, reduced cardiac function and finally heart failure (5). Thus, neutrophils can acquire also anti-inflammatory functions, which contribute to the repair and adaptation processes after AMI.

Moreover, neutrophils, like macrophages, can acquire different phenotypes, and can be divided in at least pro-inflammatory N1, and reparative N2 neutrophils (6, 7). This concept was extended by recent single-cell RNA sequencing (ScRNA-seq) studies, which demonstrated several distinct neutrophil populations (8–10), and multiple studies show a change in neutrophil phenotype on different days post MI (9–11). Therefore, altering neutrophil phenotype might be a therapeutic strategy to improve cardiac function after MI.

Insulin-like growth factor 1 (IGF1) is a key regulator of cell proliferation and survival, differentiation and metabolism and has a high homology to insulin. Signal transduction after receptor binding is believed to be highly similar for both the insulin and IGF1 receptors (IGF1R) involving the canonical PI_3_ kinase/AKT and the RAS/RAF/ERK pathways. IGF1 and insulin may also activate the non-homologous receptors, albeit at higher concentration and even hybrid receptors may be formed between IGF1 and insulin receptors. However, despite the close relationship, insulin has mainly metabolic effects, whereas IGF1 primarily functions as a growth factor. Notably, it was demonstrated recently that the preferred mitogenic effects induced by the IGF1 receptor can be explained to a large extent by structural differences residing in the intracellular juxta-membrane region of the insulin and IGF1 receptors (12).

In the context of AMI, results from both human and animal studies show that IGF1 has a high cardioprotective potential. Epidemiologic studies show that there is an inverse correlation between coronary heart disease and plasma levels of IGF1 (13). In addition, in AMI patients, low IGF1 plasma levels are associated with increased all-risk mortality, stroke, and recurrent MI (14). In mice, chronic overexpression of IGF1 significantly reduced scar formation and improved cardiac function after permanent occlusion of the left coronary artery (15). Also, in rat and swine IGF1 application before or during ischemia appears to preserve cardiac function (16, 17). On the mechanistic level, we have shown that short term, three day treatment with IGF1, starting at the end of ischemia, significantly reduces scar size, increases vascularization and improves cardiac function after MI by modulating myeloid cells. Indeed, IGF1 polarized macrophages to an anti-inflammatory M2-like phenotype both *in vitro* and *in vivo* 3 days after MI (18), leading to the hypothesis that IGF1 promotes reparative macrophage populations which supports the healing phase following MI.

In this study, we investigated the effects of IGF1 on neutrophils *in vitro* and in the context of AMI *in vivo*. We show that IGF1, but not insulin, is capable of creating an anti-inflammatory N2 phenotype in bone marrow derived neutrophils by non-canonical signaling *via* the JAK-STAT pathway. Single cell RNA-sequencing reveals that IGF1 treatment also attenuates the pro-inflammatory phenotype in neutrophils and macrophages 3 days after MI demonstrating that IGF1 acts as a neutrophil modulating cytokine.

## Results

### IGF1 and the N2 Polarizer IL4 Induce Almost Identical Transcriptional Changes in Neutrophils

**Figure 1:**
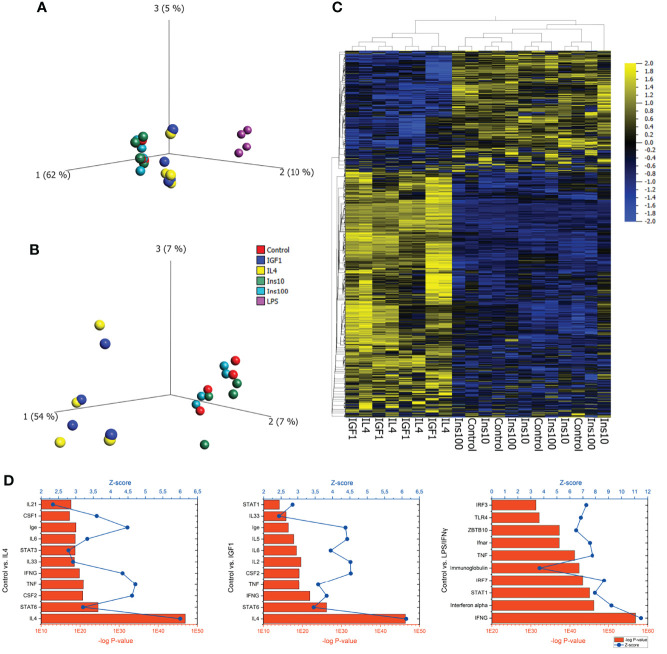
IGF1 and IL4 induce almost identical transcriptional changes in neutrophils **(A)** PCA plot of bone marrow derived neutrophils, untreated or treated for 4 hours with LPS/IFNγ (10/2 ng/mL), IL4 (20 ng/mL), IGF1 (10 ng/mL) or insulin (10 ng/mL and 100 ng/mL) after RNAseq analysis of transcriptomes shows 3 clusters of neutrophils. **(B, C)** A PCA plot **(B)** and a hierarchical clustered heatmap **(C)** without LPS/IFNγ treated neutrophils shows that IL4 and IGF1 treated, as well as control and insulin treated neutrophils cluster together. **(D)** IPA analysis of control vs IL4, IGF1 and LPS/IFNγ treated neutrophils identified upstream regulators. Bars represents –log p-values, line represents Z-scores.

To assess the effect of IGF1 on neutrophil phenotype, bone marrow derived neutrophils were treated for 4 hours with the N1 polarizers LPS/IFNγ, the N2 polarizer IL4, or IGF1 or insulin. RNAseq analysis of neutrophil transcriptomes was performed to obtain information on the neutrophil phenotypes after the different treatments. LPS/IFNγ treatment changed 2827 genes when compared to untreated neutrophils, of which 872 were upregulated and 1957 were downregulated. Interestingly, IL4 and IGF1 treatment led to a similar quantitative modification of gene expression (IL4: 286 genes, 261 up- and 25 downregulated, IGF1: 303 genes, 264 up- and 39 downregulated compared to untreated cells). Unexpectedly, insulin, even though it activates the same pathways as IGF1, had almost no effect on gene transcription neither after treatment with a physiological dose (10 ng/mL), nor after high-dose treatment (100 ng/mL). Principal component analysis and hierarchical clustering showed that IL4 and IGF1 treated neutrophils formed the same cluster (**Figures 1A–C**), as did untreated and insulin treated neutrophils. In addition, when comparing altered genes between IL4 and IGF1 treated neutrophils, none were found to be differentially expressed, whereas between IGF1 and insulin 217 and 241 up- and downregulated genes were observed respectively for physiological and high dose insulin. Ingenuity Pathway Analysis (IPA) to identify upstream regulators (**Figure 1D**) showed that pro-inflammatory regulators were the most upregulated after LPS/IFNγ treatment (interferon γ p<10^-57^, Z-score 11.5, STAT1 p<10^-44^, Z-score 7.9). After IL4 treatment, the highest upregulated upstream regulators were IL4 (p< 10^-46^, Z-sore 5.9) and the canonical downstream target STAT6 (p< 10^-23^, Z-score 3.2). Unexpectedly, after IGF1 treatment regulators well known to be affected by IGF1 such as the PI3K/AKT and/or RAS/MAP kinase pathways were not present among the top 10 of upstream regulators. The most upregulated upstream regulators were also IL4 (p< 10^-45^, Z-sore 6.1) and STAT6 (p< 10^-25^, Z-sore 3.5). Even more, the top 10 of upstream regulators identified by IGF1 treated neutrophils was almost identical to the top 10 of IL4 treated neutrophils. These data show that IGF1 and insulin have different effects on neutrophils, whereas IL4 and IGF1 treatment causes almost identical transcriptional changes suggesting that IGF1 modulated neutrophil polarization by signaling pathways identical to IL4.

### IGF1, But Not Insulin, Polarizes Neutrophils to a N2-Like Phenotype

**Figure 2:**
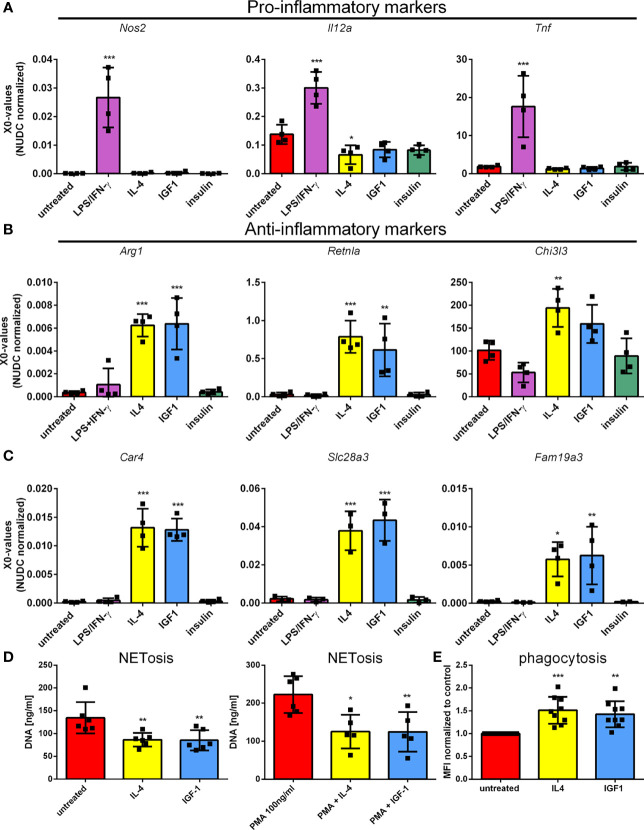
IGF1, but not insulin, polarizes neutrophils to a N2-like phenotype Bone marrow derived neutrophils were left untreated or treated 4 hours with LPS/IFNγ (10/2 ng/mL), IL4 (20 ng/mL), IGF1 (10 ng/mL) or insulin (10 ng/mL) and pro-inflammatory marker **(A)**, anti-inflammatory marker **(B)**, and the highest upregulated genes observed in RNAseq **(C)** expression was assessed by quantitative RT-PCR. **(D)** DNA content in supernatant was measured as a measure for NETosis, in the absence and presence of 100 ng/mL phorbol 12-myristate 13-acetate (PMA). **(E)** Phagocytosis of fluorescently labeled Staphylococcus aureus normalized to untreated neutrophils. LPS, Lipopolysaccharide; IFNγ, interferon-gamma; IL4, Interleukin 4; IGF1, Insulin-like growth factor 1; *Nos2*, nitric oxide synthase 2; *Il12a*, Interleukin 12a; *Tnf*, Tumor necrosis factor; *Arg1*, Arginase 1; *Retnla*, resistin like alpha; *Chi3l3*, chitinase-3-like 3. X0 values normalized to *Nudc* (nudC nuclear distribution protein) expression of single measurements are shown. Bars represent mean ± SD. *p<0.05, **p<0.01, ***p<0.001.

To confirm the results obtained by RNAseq, qPCR was performed on genes observed to be differently regulated in RNAseq. First genes associated with pro-, and anti-inflammatory polarization were studied. LPS/IFNγ induced the expression of pro-inflammatory genes, type II nitric oxide synthase (*Nos2*), interleukin 12a (*Il12a*) and tumor necrosis factor α (*Tnf*) (**Figure 2A**), whereas IL4 induced expression of anti-inflammatory genes, arginase I (*Arg1*), resistin-like α (*Retnla*) and chitinase-3- like 3 (*Chi3l3*) (**Figure 2B**). In addition, IL4 treatment reduced *Il12a* expression. As for macrophages, IGF1 treatment promoted the development of an anti-inflammatory phenotype in neutrophils, characterized by the elevated expression of anti-inflammatory marker genes to almost the same extent as IL4. As observed in RNAseq, insulin was unable to increase expression of either anti-, or pro-inflammatory genes. In addition, the genes that were upregulated the most after IGF1 and IL4 treatment, carbonic anhydrase 4 (*Car4*), Solute carrier family 28 member 3 (*Slc28a3*) and the known M2 marker gene Family with sequence similarity 19, member A3 (*Fam19a3*). All 3 markers confirmed the results of RNAseq and were upregulated in both IL4 and IGF1 treated neutrophils, but not after insulin or LPS/IFNγ treatment (**Figure 2C**). These result show that IGF1, but not insulin creates an anti-inflammatory phenotype in neutrophils. To analyze if the transcriptional changes translate into an anti-inflammatory phenotype of neutrophils after IGF1 treatment functional assays were applied. Neutrophils are known to release neutrophil extracellular traps (NETs) that are composed of extruded DNA. Both IL4 and IGF1 treatment of unstimulated neutrophils significantly reduced the amount of dsDNA in medium with 35.9 and 36.4% respectively (**Figure 2D**), but even stronger after stimulation with PMA (43.8 and 44% for IL4 and IGF1, respectively). Another function of neutrophils is phagocytosis. Increased phagocytosis is a characteristic of M2 macrophages (19). Both IL4 and IGF1 treatment increased phagocytosis of fluorescently labeled Staphylococcus aureus by 51 ± 3% and 43 ± 3%, respectively (**Figure 2E**). These data indicate that IGF1 treatment also alters the function of neutrophils to be more anti-inflammatory.

### Neutrophil Polarization Is Not Caused by Activation of the Known IGF1 Pathway

**Figure 3:**
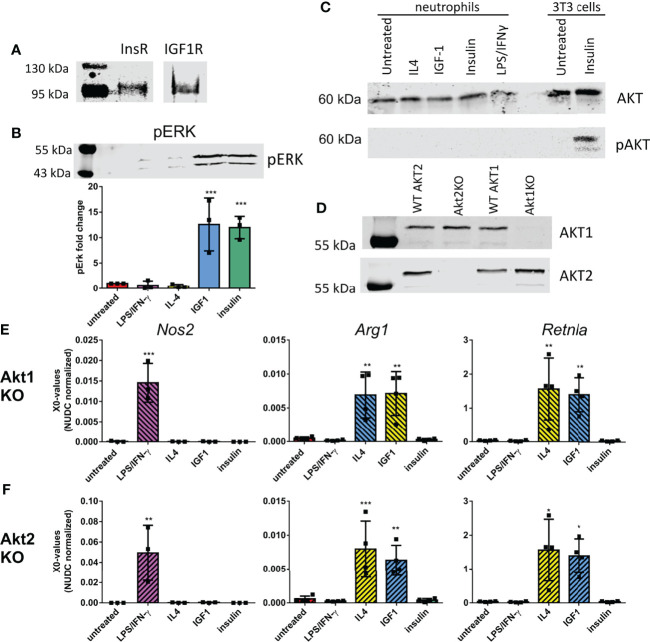
Neutrophil polarization is not caused by activation of the known IGF1 pathways **(A)** Immunoprecipitation showed the presence of the insulin-like growth factor 1 receptor (IGF1R) and insulin receptor (InsR) in bone marrow derived neutrophils **(B)** 10 min IGF1 and insulin treatment caused phosphorylation of ERK (pERK) as shown by western blot. Data is normalized to pERK in untreated neutrophils. **(C)** Western blot analysis showed the presence of AKT in bone marrow derived neutrophils, however none of our treatments caused AKT phosphorylation (pAKT) after 10 min treatment. As a positive control AKT phosphorylation of 3T3 cells after insulin treatment is shown on the same western blot. **(D)** Akt1 and 2 expression in Tie2-Cre Akt1^fl/fl^ and Tie2-Cre Akt2^fl/fl^ mice was assessed by Western blot. **(E-F)** N1 and N2 polarization after our treatments was unaffected in Tie2-Cre Akt1^fl/fl^ **(E)** and Tie2-Cre Akt2^fl/fl^ **(F)** neutrophils as shown by normal upregulation in quantitative RT-PCR of *Nos2* after LPS/IFNγ treatment and *Arg1* and *Retnla* after IL4 and IGF1 treatment. LPS: Lipopolysaccharide, IFNγ: interferon-gamma, IL4: Interleukin 4, IGF1: Insulin-like growth factor 1, *Nos2*: nitric oxide synthase 2, *Arg1*: Arginase 1, *Retnla*: resistin like alpha. X0 values normalized to *Nudc* (nudC nuclear distribution protein) expression of single measurements are shown. Bars represent mean ± SD. *p<0.05, **p<0.01, ***p<0.001.

Since insulin and IGF1 treatment have different effects on neutrophil polarization and RNAseq data did not lead to identification of IGF1/insulin signaling as the main upstream regulator causing the observed changes, we investigated if both receptors are expressed by neutrophils. Immunoprecipitation showed that both the insulin and IGF1 receptors were present on neutrophils (**Figure 3A**). IGF1 and insulin are known to both activate the RAS/MAPkinase and the PI3K/AKT pathways. To study the involvement of these pathways in neutrophil polarization, we first looked at ERK phosphorylation. Treatment of neutrophils with insulin or IGF1 enhanced phosphorylation of the downstream protein ERK, whereas LPS/IFNγ and IL4 treatment did not (**Figure 3B**). We also investigated AKT phosphorylation. Interestingly, although AKT is expressed by neutrophils, none of our treatments was able to induce phosphorylation of AKT (**Figure 3C**). To directly assess an AKT involvement in polarization, neutrophils from Tie2-Cre Akt1^fl/fl^ and Tie2Cre Akt2^fl/fl^ knock-out mice were treated with the different polarizers. Tie2-Cre mice, which were developed to study transgenic expression in endothelial cells, have been shown to also affect hematopoietic cells (20). Western blot and qPCR analysis showed that in Tie2-Cre Akt1^fl/fl^ mice, Akt1 protein and gene expression were absent, without affecting Akt2, and in Tie2-Cre Akt2^fl/fl^ mice Akt2 expression was absent, without affecting Akt1 (**Figures 3D** and **S1**).

The knock-out of *Akt1* or *Akt2* did not affect the upregulation of the N1 marker *Nos2* after LPS/IFNγ treatment (**Figures 3E, F**). Also the IL4 and IGF1 induced upregulation of the N2 markers *Arg1* and *Retnla* were not affected by the isotype specific AKT knock-out (**Figures 3E, F**), indicating that neutrophil polarization by IGF1 is independent of either AKT1 or AKT2.

### IGF1 Induces Neutrophil Polarization *via* JAK Activation

**Figure 4:**
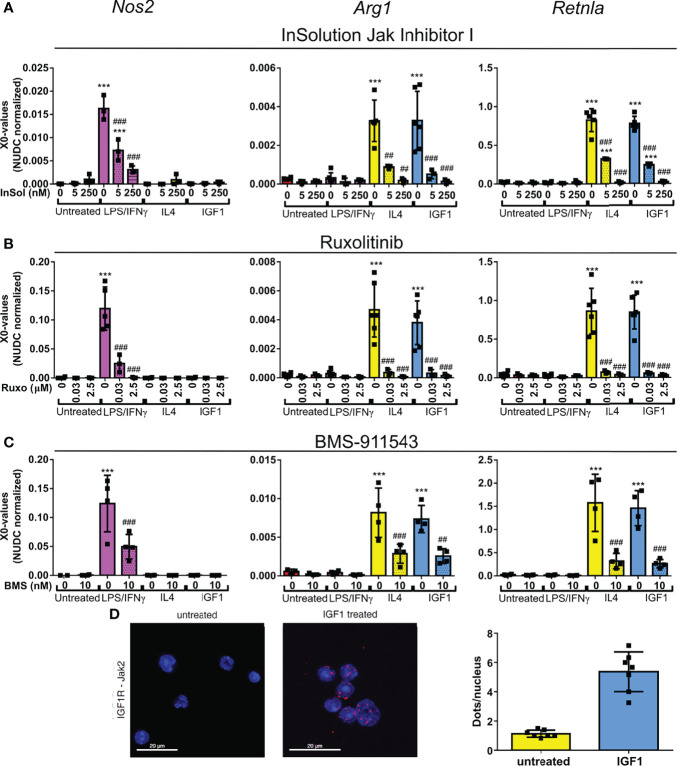
IGF1 induces neutrophil polarization *via* JAK activation **(A–C)** Quantitative RT-PCR analysis of bone marrow derived neutrophils untreated, or treated for 4 hours with LPS/IFNγ (10/2 ng/mL), IL4 (20 ng/mL) or IGF1 (10 ng/mL) in the presence or absence of the JAK inhibitors InSolution Jak Inhibitor 1 (Insol) **(A)**, Ruxolitinib (Ruxo) **(B)** or BMS-911543 (BMS) **(C)** shows that neutrophil polarization is dependent on Jak activation. EC_50_ values of the JAK inhibitors are shown in **Table 1**. X0 values normalized to *Nudc* (nudC nuclear distribution protein) expression of single measurements are shown. **(D)** Representative images and analysis of untreated and IGF1 treated neutrophils after proximity ligation assay with JAK2 and IGF1 receptor (IGF1R). Proximity ligation assay analysis of IGF1R with JAK1, JAK3 and TYK2 are shown in **Figure S1**. LPS, Lipopolysaccharide; IFNγ, interferon-gamma; IL4, Interleukin 4; IGF1, Insulin-like growth factor 1; *Nos2*, nitric oxide synthase 2; *Arg1*, Arginase 1; *Retnla*, resistin like alpha. Single measurements are shown. Bars represent mean ± SD. *p<0.05, **p<0.01 and ***p<0.001 when compared to untreated without inhibitor. ^#^p<0.05, ^##^p<0.01 and ^###^p<0.001 when compared to the same treatment without inhibitor.

For macrophages it is known that polarization to a M1 or M2 phenotype after LPS/IFNγ or IL4 treatment depends on activation of the JAK/STAT pathway. For neutrophils this is still unknown. The almost identical transcriptional changes in IGF1 and IL4 treated neutrophils prompted us to take a closer look at this pathway using a combination of JAK inhibitors. InSolution™ JAK Inhibitor I has an IC_50_ value for JAK1 that is higher than that of the other JAKs and TYK2 (**Table 1**). Treatment of neutrophils with a high dose (250 nM) of InSolution™ JAK Inhibitor I, inhibiting all JAKs and TYK2, reduced LPS/IFNγ induced upregulation of *Nos2* and prevented IL4 induced upregulation of anti-inflammatory genes (**Figure 4A**). Notably, also IGF1 was no longer capable of polarizing neutrophils to an anti-inflammatory phenotype, indicating that JAK activation is necessary for IGF1 induced N2 polarization. At a low dose (5 nM), at which only JAK1 is active, InSolution™ JAK Inhibitor I prevented a significant upregulation of *Arg1* after both IL4 and IGF1 treatment and reduced the upregulation of *Retnla* by 61% and 69% for IL4 and IGF1, respectively. LPS/IFNγ induced upregulation of pro-inflammatory *Nos2* was significantly reduced by 65%, but was still significantly higher than in untreated cells. This shows that JAK1 appears to contribute to IL4 and IGF1 induced N2 polarization and might be necessary for upregulation of pro-inflammatory genes after LPS/IFNγ. To narrow down which other JAK isoform is responsible for neutrophil polarization, we used the JAK inhibitor Ruxolitinib, which has a 150 times lower IC_50_ for JAK1/2 than for JAK3 (**Table 1**). Again, a high concentration of 2.5 µM prevented polarization to a N1 or N2 phenotype (**Figure 4B**). A lower concentration (30nM), at which only JAK3 is active, the expression of pro- and anti-inflammatory markers after LPS/IFNγ and IL4 and IGF1 treatment, respectively, was suppressed, indicating that JAK3 activity is not necessary for either N1 or N2 polarization. Finally, inhibition of only JAK2, using BMS-911543 (IC_50_ values, see **Table 1**), prevented a significant upregulation of N1 and N2 markers (**Figure 4C**). The combined data of JAK inhibitor experiments indicate that both N1 and N2 polarization are mainly JAK2 dependent with also some contribution of JAK1.

**Table 1:** IC50 values for the Jak inhibitors used Table showing the IC50 values for the different JAK inhibitors used in this study and the concentrations used.

	IC50-values (nM)						
Inhibitor	JAK1	JAK2	JAK3	TYK2	High conc (nM)	Low conc (nM)	Inhibited at low concentration
Insolution™	15	1	5	1	250	5	JAK2/3/TYK2
Ruxolitinib	3.3	2.8	>390		2500	30	JAK1/2
BMS-911543	75	1.1	360	66		10	JAK2

The high concentration (high conc) inhibits all JAKs and TYK2, whereas at the lower concentrations (low conc) only specific JAKs are inhibited, which are shown in the last column.

JAKs are activated by phosphorylation after recruitment to activated receptors. In line with the proposed involvement of JAK2 in IGF1-mediated neutrophil polarization, proximity ligation assays with IGFR- and JAK2-specific antibodies revealed that IGF1 treatment substantially increased signal numbers when compared to unstimulated cells (**Figure 4D**). This indicates that the activated IGF1R recruits JAK2. In addition, no interaction of JAK1, JAK3 or TYK2 was observed after IGF1 treatment (**Figure S2**).

### LPS/IFNγ Cause STAT1 Phosphorylation, Whereas IL4 and IGF1 Cause STAT6 Phosphorylation

**Figure 5:**
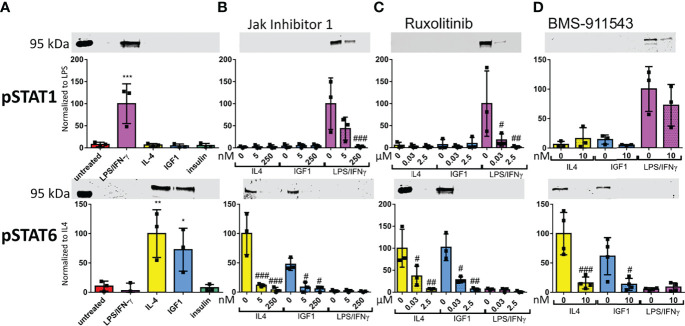
LPS/IFNγ cause STAT1 phosphorylation, whereas IL4 and IGF1 cause STAT6 phosphorylation **(A–D)** Representative western blots and analysis for STAT1 and STAT6 phosphorylation after 10 min treatment with LPS/IFNγ (10/2 ng/mL), IL4 (20 ng/mL) or IGF1 (10 ng/mL) without JAK inhibitor **(A)**, or in the presence of different concentrations of InSolution Jak Inhibitor 1 **(B)**, Ruxolitinib **(C)** or BMS-911543 **(D)**. Data is normalized to total protein stain, and for pSTAT 1 LPS/IFNγ without inhibitor is set to 100 and for pSTAT6 IL4 without inhibitor is set to 100. Single measurements are shown. Bars represent mean ± SD. *p<0.05, **p<0.01 and ***p<0.001 when compared to untreated without inhibitor. ^#^p<0.05, ^##^p<0.01 and ^###^p<0.001 when compared to the same treatment without inhibitor.

STAT1 and 6 phosphorylation downstream of activated JAK is known the be associated with M1 and M2 polarization in macrophages, respectively (21, 22). Therefore, we studied the effect of our treatments on STAT1 and STAT6 phosphorylation levels in neutrophils. STAT1 was phosphorylated in response to LPS/IFNγ only (**Figure 5A**). STAT6 was phosphorylated after both IL4 and IGF1 treatment (**Figure 5A**). Insulin treatment had no effect on STAT activation. When only JAK 1 or JAK 3 were active after treatment with Insolution™ JAK Inhibitor I or Ruxolitinib, respectively, no STAT6 phosphorylation was observed after IL4 and IGF1 treatment (**Figures 5B, C**), indicating that JAK2 activation is necessary for IL4 and IGF1 mediated STAT6 activation. Indeed, inhibition of JAK2 with BMS-911543, prevented STAT6 phosphorylation after IL4 and IGF1 treatment (**Figure 5D**). STAT1 phosphorylation after LPS/IFNγ treatment, was absent when JAK1 and 2 were inhibited with Ruxolitinib (**Figure 5C**). JAK1 activity alone, was sufficient to cause STAT1 phosphorylation, although less than without inhibition of the other JAKs (**Figure 5B**). JAK2 inhibition had no effect on STAT1 phosphorylation after LPS/IFNγ treatment (**Figure 5D**). These results show that JAK1 mediates STAT1 activation after LPS/IFNγ treatment, whereas JAK2 is responsible for STAT6 activation after IL4 and IGF1 treatment.

### IGF1 Treatment Reduces the Pro-Inflammatory Phenotype of Neutrophils After MI

**Figure 6:**
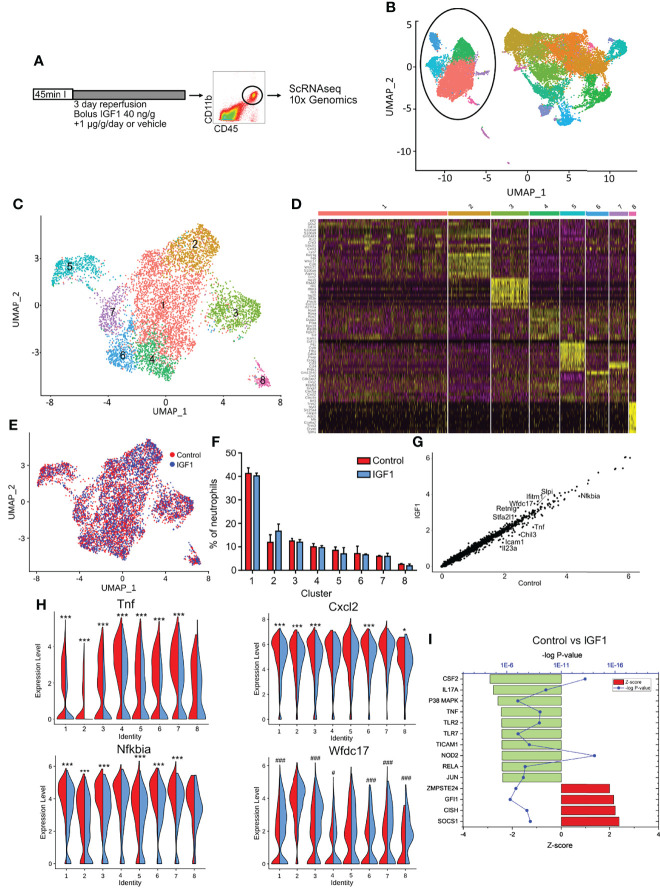
IGF1 treatment creates an anti-inflammatory phenotype of neutrophils after myocardial infarction. **(A)** Experimental protocol. C57BL/6J mice were subjected to 45-min LAD coronary artery occlusion and 3 days of reperfusion. At the start of reperfusion mice were treated with IGF1 or vehicle for 3 days. Hearts were digested and cardiac CD45+CD11b+ cells were sorted out for single cell RNA sequencing using the 10x Genomics platform. **(B)** UMAP of all CD45+CD11b+ cells shows a clear difference between neutrophils and other CD11b+ cells. Neutrophils are encircled. **(C)** Reclustering of neutrophils with a higher resolution results in 8 different neutrophil clusters. **(D)** Heatmap depicting the top 10 differently expressed genes for each cluster. **(E)** UMAP showing control (red) and IGF1 treated (blue) neutrophils does not show a differences in the distribution of neutrophils over the clusters. **(F)** The amount of neutrophils per cluster as percentage of the total number of neutrophils. **(G)** A scatter plot of all pooled neutrophils shows differences in gene expression between control and IGF1 treated neutrophils. The top 10 differently expressed genes are shown. **(H)** Violin plots show a clear difference in the expression of *Tnf*, *Nfkbia, Cxcl2* and *Wfdc17* in most clusters between control (red, left) and IGF1 (blue, right) treated neutrophils. **(I)** Upstream regulators that are affected after IGF1 treatment with a Z-score>2.0 after pooling all neutrophils. The 12 most changed upstream regulators are shown. Upstream regulators per cluster are represented in **Figure S4**. *p<0.05 higher in control, ***p< 0.001 higher in control, ^#^p<0.05 higher in IGF1, ^###^p<0.001 higher in IGF1.

Previously we could show that IGF1 improves outcome after MI by modulation of myeloid cells (18). To investigate to what extent IGF1 also creates an anti-inflammatory phenotype in neutrophils and macrophages *in vivo* we exposed mice to MI followed by reperfusion with and without IGF1 treatment and performed single cell RNA-sequencing (scRNA-seq) of cardiac myeloid cells, 3 days post-MI (**Figure 6A**). As demonstrated before, IGF1 treatment was protective and improved ejection fraction, stroke volume and cardiac output 1 week post-MI (**Figure S3**). Transcriptional profiling of all non-myocyte cells of the adult mouse heart performed by others focused on macrophage and fibroblast populations and resulted in none or only one granulocyte cell cluster (23, 24). In order to improve resolution of neutrophils, we isolated myeloid cells (CD45+CD11b+) by FACS sorting before scRNA-seq. Cell clustering with low resolution showed a clear distinction between neutrophils and other myeloid cells (monocytes, macrophages, dendritic cells) (**Figure 6B**). Both subsets were then reclustered with a higher resolution. The neutrophil subset contained in total 6635 cells, 3533 from control and 3102 from IGF1 treated mice. In neutrophils a medium of 2415 transcripts per cell was detected. The number of transcripts per cell was equal in the control and IGF1 treated groups. Unsupervised clustering created 8 cardiac neutrophil subsets (**Figure 6C**). Cluster NC1 is the largest neutrophil cluster and is characterized by a higher expression of, amongst them *Klf2*, *G0s2* and *Cd14* (**Figure 6D**). Neutrophils in cluster NC2 may represent young blood derived neutrophils characterized by the expression of transcripts such as *Retnlg, Slpi* and *Wfdc17* (10), indicating that these neutrophils might have entered the heart shortly before isolation. Cluster NC3 neutrophils express a high level of type I interferon-stimulated genes (e.g. *Isg15, Rsad2, Ifit1*). Cluster NC4 has a high expression of transcripts encoding ribosomal genes (*Rps8, Rpsa, Rps19, Rpl21*) and, compared to the other clusters, a significantly higher expression of, amongst others, *Tnf, Dusp2, Ppia, Icam1* and *Siglecf*. This transcription pattern is characteristic for old neutrophils, that have a more pro-inflammatory phenotype (9, 10). Cluster NC5 is characterized by the expression of genes including *Gdf15*, *Ftl1* and *Cstb*, and the absence of *Cxcr2*. Cluster NC6 and NC7 seem to be quite similar concerning gene expression. The main difference is a high expression of *Gm12840* in cluster NC6, whereas neutrophils in cluster NC7 have a high expression of *Ccl3* and *Ccl4*. Transcripts specific for cluster NC8, are genes associated with cardiomyocytes (e.g. *Tnnt2*, *Myl3*, *Actc1*, *Mb*). However, these cells express neutrophil markers (*S100a8*, *S100a9, Csf3r, Cxcr2, Mmp9, Csf1* and *Il1r2*) similar to the other clusters. They also have a comparable number of genes and number of transcripts indicating that these are not doublets (**Figure S4A**). Therefore, we assume that these neutrophils had phagocytosed other cells, which could explain the presence of cardiomyocyte derived transcripts.

The number of neutrophils per cluster was similar for each replicate, showing little variation between experiments. Analysis of cell distribution over the different clusters with and without IGF1 treatment, and the percentages of neutrophils in each cluster, revealed no clear differences (**Figures 6E, F**), indicating IGF1 does not affect cell distribution over the clusters. However, a scatter plot of all neutrophils, with control and IGF1 neutrophils on the x- and y- axis, respectively, indicated that IGF1 treatment downregulates *Nfkbia*, *Tnf*, *Icam1*, *IL23a* and *Chil3* and upregulates *Retnlg*, *Wfdc17, Ifitm1*, *Stfa2l1* and *Slpi* expression (**Figure 6G**). To see which clusters of neutrophils were responsible for this effect, we performed differential gene expression analysis within each cluster between IGF1 and control neutrophils (**Table S1**). We observed that IGF1 treatment downregulated genes involved in NFκB signaling in most clusters. *Tnf* expression was reduced in all clusters, except cluster 8, and *Nfkbia* and *Icam1* were downregulated in 6 out of 8 clusters (**Figures 6H** and **S4B**). On the other hand, *Wfdc17* and *Slpi* (**Figures 6H** and **S4B**), which have been shown to inhibit NFκB activity and reduce inflammation (25–28) are upregulated in 6 and 5 of 8 clusters, respectively.

To get more quantitative measures for the IGF1 effects we used the upstream regulator function in IPA to identify possible regulators that might have caused the alterations in gene expression patterns. When we used the DGE of all neutrophils (**Table S1**), IPA analysis showed a downregulation of NFκB involved upstream regulators. TNFα and NFκB subunit Rela were downregulated after IGF1 treatment (p=10^-10^, Z-score 2.45 and p=10^-8^, Z-score 2.4, respectively) (**Figure 6I**) and the NFκB pathway itself was downregulated in 4 of 8 clusters (**Figure S4C**). Furthermore, the upstream regulators TLR2 (p=10^-9^, Z-score 2.4), TLR7 (p=10^-8^, Z-score 2.4), TICAM1 (p=10^-9^, Z-score 2.4) and NOD2 (p=10^-15^, Z-score 2.4), which are all known to activate NFκB, were downregulated after IGF1 treatment (**Figure 6I**). The pathway that was downregulated with the highest Z-score (2.9, p=10^-14^) was the granulocyte-macrophage colony stimulating factor (CSF2) (**Figure 6I**). In addition, *Cxcl2*, the potent chemokine responsible for the attraction of neutrophils, was downregulated in 5 of 8 clusters after IGF1 treatment (**Figure 6H**). These markers indicate a reduction in neutrophil production and neutrophil chemotaxis respectively. IGF1 treatment upregulated the SOCS1 pathway (p=10^-9^, Z-score 2.4) and Cytokine-inducible SH2-containing protein (CISH) pathway (p=10^-8^, Z-score 2.2). Both have been associated with a reduction of pro-inflammatory cytokines (29, 30). Indeed, we observed a reduction in gene expression of *Tnf* and *Il23a*, and a downregulation of the TNF, IL17a and IL1β pathway after IGF1 treatment (**Figures 6H, I**, **S4C** and **Table S1**)

Together, these results show that IGF1 treatment after MI does not create a specific type of neutrophil, or changes the distribution of neutrophils over the clusters, but reduces the pro-inflammatory phenotype of neutrophils in each cluster.

### IGF1 Treatment Reduces the Pro-Inflammatory Phenotype in Macrophages After MI

Also the other myeloid cells (monocytes, macrophages and dendritic cells) were re-clustered. This subset contained 14143 cells, 6870 in the control group and 7273 in the IGF1 treated group, with a median of 13 021 transcripts per cell. Reclustering resulted in 15 clusters, with 1 cluster of dendritic cells (DC) (CD209 DC; cluster MC8), 1 cluster of monocytes (MC14), 1 cluster of monocytes turning into macrophages (MC2) and 12 macrophage clusters (**Figures S5A, B**). These clusters were comparable to clusters observed after MI by Dick et al. (31). Cluster MC9 was comparable to the Timd4 cluster, with a characteristic expression of *Timd4* and *Lyve1*, cluster MC12 with the MHCII cluster, with a higher expression of antigen-presentation genes, cluster MC7 were CCR2+ macrophages, cluster MC5 was enriched in type I interferon-stimulated genes, cluster MC6 are proliferating macrophages and the other clusters are macrophages specific to MI as defined by Dick et al. (31). Also for macrophages, IGF1 treatment did not alter the distribution of cells over the clusters (**Figures S5C, D**), but in general showed attenuated expression of pro-inflammatory genes (**Table S2**). Also here, the top 5 of downregulated genes were pro-inflammatory; *Nfkbia* was downregulated in 8, *Tnf* and *Cxcl2* in 6, and *Dusp2* and *Tnfsf9* in 4 clusters (**Figures S5E, F**). The top 5 of upregulated genes after IGF1 treatment included *Hspa8* (10 clusters), *Hspa5* and *C1qa* (9 clusters) and *C1qc* and *C1qb* (7 clusters) (**Figures S5E, F**). *C1q* has been associated with M2 polarization of macrophages (32). IPA analysis of DGE after IGF1 treatment in each cluster, also showed a reduced inflammatory type, with downregulation of the pro-inflammatory upstream regulators TNF, IL1β, MyD88 and TICAM1 and an upregulation of anti-inflammatory CISH (clusters MC 3, 4, 5, 10, 11 and 13), whereas others showed the opposite (clusters MC8, 9, 12 and 15) (**Figure S5G**). Thus, the pattern of reduced pro-inflammatory gene expression was also found in macrophages, although not as pronounced as for the neutrophils.

## Discussion

We show that IGF1 polarizes neutrophils to an anti-inflammatory phenotype *in vitro* by activation of the non-canonical downstream JAK-STAT pathway. Interestingly, insulin, which is highly similar to IGF1, does not activate this pathway, and has no effect on neutrophil polarization. 3 day IGF1 treatment after MI improved cardiac function and attenuated the expression of pro-inflammatory genes in neutrophils and macrophages as shown by scRNA-seq of myeloid cells isolated from mouse hearts. Thus, IGF1 acts like an anti-inflammatory cytokine *in vitro* and *in vivo*.

Comprehensive transcriptional profiling of all non-myocyte cells of the adult mouse heart only results in 1 granulocyte population (23, 24). Therefore, we isolated myeloid cell by FACS sorting before scRNA-seq. ScRNA-seq showed that IGF1 treatment after MI did not create a specific type of neutrophil or macrophage, because no specific “IGF1 cluster” was detected. In addition, the distribution of neutrophils and macrophages over the clusters was quantitatively similar for IGF1 and control cells, indicating that IGF1 did not affect the size of a specific cluster. However, when looking at differences between control and IGF1 treated cells within each cluster, a clear IGF1 effect was observed for neutrophils, and to a lesser extent in macrophages. In most clusters, IGF1 downregulated pro-inflammatory genes, attenuated effects of pro-inflammatory upstream regulators, and enhanced contribution of anti-inflammatory upstream regulators based on IPA analysis of differently expressed genes.

A major target of IGF1 treatment *in vivo* appeared to be NF-κB, because NF-κB related genes including *Tnf*, *Icam1*, and the NF-κB-negative feedback gene *Nfkbia* were less expressed in both IGF1-treated neutrophils and macrophages after MI. In addition, an upregulation of *Wfdc17* and *Slpi* and *C1q* was observed in IGF1 treated neutrophils and macrophages, respectively, which have been associated with inhibition of NF-κB activity (25–27, 33, 34). Also IPA analysis of possible upstream regulators indicated a reduced activity of regulators that are known to activate NF-κB, including TLR2, TLR7, TICAM1 and NOD2. The NF-κB pathway plays an important role in cells of the innate immune system. It is responsible for the transcriptional induction of pro-inflammatory cytokines, chemokines and inflammatory mediators (35). In addition, these mediators can promote differentiation of inflammatory T-cells. In macrophages, NF-κB is a key transcription factor for M1 macrophages and required for the induction of pro-inflammatory genes (36). In neutrophils, NF-κB activation mediates cell adhesion, promotes inflammation, inhibits neutrophil apoptosis and has been linked to NETosis (37). Of note, NF-κB-DNA binding activity was higher in IGF1R knock-out macrophages, and the enhanced production of TNFα and IL6 in IGF1R KO macrophages after IFNγ treatment was completely abolished by NF-κB inhibitors (38).

Next to genes involved in the NF-κB pathway, IPA analysis showed an upregulation of SOCS1 and CISH. Both have been associated with a reduction in pro-inflammatory cytokines (29, 30). Indeed, we observed a reduction in gene expression of *Tnf* and *Il23a*, and a downregulation of the TNF, IL17a and IL1β pathway after IGF1 treatment. Furthermore, we observed a downregulation of cysteine/glutamate transporter *Slc7a11* in IGF1 treated neutrophils. *Slc7a11* has been associated with ROS production (39). Additionally, IGF1 treatment upregulated genes that enable endopeptidase inhibitor activity (*Wfdc17, Stfa2l1* and *Slpi*). Neutrophil endopeptidases are able to destroy the extracellular matrix, and therefore their inhibition could be cardioprotective. Indeed, several studies show a reduction in ischemia-reperfusion injury after endopeptidase inhibition (40, 41). Moreover, IGF1 treatment caused a downregulation of the expression of the chemokine *Cxcl2* in both neutrophils and macrophages. Cxcl2 is a potent chemokine released by neutrophils and macrophages, responsible for attracting more neutrophils (42). IPA analysis also showed a downregulation of CSF2 in neutrophils after IGF1 treatment. CSF2, also known as granulocyte-macrophage colony stimulating factor, stimulates growth and proliferation of different hematopoietic precursor cells, including neutrophils and macrophages. A reduction in neutrophil chemotaxis to the infarcted area and reduced production of neutrophils and macrophages might indicate resolution of inflammation. Recent scRNA-seq studies of cardiac neutrophils after MI identified neutrophils with a high expression of SiglecF, which were pro-inflammatory (9, 10). Interestingly, differentially expressed genes in our IGF1 treated neutrophils seem to be regulated in an opposite direction of genes that contributed to the pro-inflammatory phenotype in SiglecF^HI^ neutrophils (9). Of the top 10 genes that were downregulated in SiglecF^HI^ neutrophils, 6 were upregulated after IGF1 treatment in at least 1 cluster (*Retnlg, Slpi, Ccl6, Asprv1* and *Lrg1*), and of the top 10 genes that were upregulated in SiglecF^HI^ neutrophils, 4 were downregulated after IGF1 treatment in at least 1 cluster (*Hexb, Nfkbia, Icam1* and *Tnf*). Altogether, this shows that IGF1 treatment attenuates the pro-inflammatory phenotype in neutrophils and in macrophages.

Using qPCR, FACS analysis and aptamer proteomics others showed that the amount of anti-inflammatory neutrophils increases with time after MI (7, 11). Our scRNA-seq results show that these anti-inflammatory neutrophils do not form a specific cluster, but that the neutrophils in existing clusters become less inflammatory. Also others that performed scRNAseq on different days after MI did not find a specific cluster of anti-inflammatory neutrophils (9, 10). Similar results were obtained for tumors (8) or bacterial infection (43). Xie et al. observed that *E. coli* challenge did not change the identity of neutrophil populations, which still had the same signature genes when compared to control neutrophils (43). However, infection up-and downregulated genes within each subpopulation, indicating that neutrophils within each cluster adapt themselves to the infection. Likewise, we and others (31) did not observe a specific M2-like cluster for macrophages post-MI using ScRNA-seq. The accumulating data on *in vivo* cell macrophage and neutrophil cell clusters supports the view that the extensive polarization induced by e.g. single cytokines is a helpful classification *in vitro*, but cannot be simply transferred to *in vivo* conditions. This finding is not unexpected because *in vivo* cells are modulated by amongst others a plethora of growth factors, cytokines and lipid mediators. For macrophages, it was demonstrated that far more phenotypes with divergent expression patterns than the classical M1/M2 macrophages can be induced, demonstrating that M1/M2 reflect only a minor part of their extensive plasticity. Thus, the combined activity of various polarizers affecting macrophages in a complex scenario as in MI, will not result in a defined M1/M2 state. Today it is unclear if neutrophil polarization shows a similar diversity in response to different polarizers. However, it can be expected that *in vivo* modulation of neutrophils by one additional cytokine (such as IGF1) will rather shift the expression patterns of neutrophils than polarizing them to a defined state as shown by scSeq data obtained by us and others.

Despite all limitations of the concept of *in vitro* polarization, this experimental approach is useful to analyze signaling pathways which are involved in the action of a specific cytokine. *In vitro*, IGF1 upregulated anti-inflammatory marker genes and the global effects of IGF1 on neutrophil gene expression as assessed by RNAseq were identical to that of IL4, a known N2 polarizer (7). In addition, functional assays show identical responses for IGF1 and IL4. Both reduce NETosis, which has been negatively correlated with outcome after MI (44), and increase phagocytosis, which is also a characteristic of anti-inflammatory M2 macrophages (19). Like IL4, IGF1 uses the JAK-STAT pathway to induce N2-like neutrophil polarization *in vitro*. Although some reports linked JAK-STAT signaling to IGF1 mediated signal transduction, this pathway is rather non-canonical in contrast to the well- established signaling *via* PI3K/AKT and the RAS/RAF/ERK pathways. Previously, it was shown that IGF1 can activate JAK1 and JAK2 and thereby phosphorylation of STAT3, but not STAT5, in IGF1 receptor overexpressing 293T cells (45). For primary neutrophils we show here that IGF1 enhances phosphorylation of STAT6. Thus, STAT6 phosphorylation, which is known to be important for IL4 induced macrophage polarization to an anti-inflammatory phenotype (21) also seems to be necessary for the polarization to an N2 phenotype. Although small molecule inhibitors with different specificities towards the various JAK isoforms have been developed, the specific inhibition of single JAK isoforms cannot be achieved because of overlapping inhibitory functions. Using several inhibitors at different concentrations to make use of different IC_50_ values we narrowed down JAK2 as the major JAK isoform mediating the effects of IL4 and IGF1 on neutrophil polarization. Of note, an inhibitor with relatively high specificity for JAK2 prevented N2 polarization of IL4 and IGF1 to a large extent and also suppressed STAT6 phosphorylation. In addition, an IGF1-inducible close spatial interaction of JAK2 and the IGF1R, supports a direct activation of JAK2 by the IGF1 receptor.

Whereas all of our data point towards a JAK2-STAT6 axis in N2 like neutrophil polarization *in vitro*, we found no indication that the IGF1 effect required AKT or ERK phosphorylation. N2-like polarization was still detected in bone marrow derived neutrophils from AKT1 and AKT2 knockout mice, and IGF1 in concentrations that induce N2-like polarization did not increase AKT phosphorylation. Moreover, insulin, although activating the RAS/RAF/ERK pathway, induced neither STAT6 phosphorylation nor N2 polarization, which argues against a substantial impact of ERK in N2-like polarization.

The functional differences between IGF1 and insulin in neutrophil polarization are an interesting aspect of the cell-type specific actions of both closely related hormones raising the question how the precise interactions of the IGF1R and JAK2 are mediated. The proximity ligation assay demonstrates a close spatial interaction of IGF1R and JAK2. However, if this requires adapter molecules remains unclear.

Polarization of neutrophils has been shown to play an important role in different diseases. Pro-tumorigenic N2 neutrophils produce pro-angiogenic factors, matrix-degrading enzymes (46, 47), and pro-metastatic proteins (48). On the other hand, anti-tumor N1 neutrophils generate growth inhibiting reactive oxygen species (49) and increase immune recognition of tumor cells (50). Thus, promotion of N1 neutrophils may be the preferred therapeutic option to fight cancer cells. In cardiovascular disease, however, a shift towards N2 neutrophils may have therapeutic benefit. In stroke, N2 neutrophils promote phagocytosis (51, 52). Thereby they increase the removal of debris from the inflamed tissue, but also cause self-clearance. This can contribute to the restoration of tissue homeostasis and improve stroke outcome. In myocardial infarction neutrophil phenotype has been shown to influence left ventricular remodeling (7). N1 neutrophils were correlated with infarct wall thinning, whereas N2 neutrophils seem to attenuate adverse left ventricular remodeling (7). We already showed that the cardioprotective effects of IGF1 after MI are caused by myeloid cells (18). This study now shows that IGF1 can attenuate the pro-inflammatory phenotype in neutrophils and macrophages after MI, which may represent the key mechanism for its cardioprotective effects.

## Material and Methods

### Animals

Experiments were performed with neutrophils isolated from C57Bl/6J mice. Mice were housed under standard housing conditions (12h dark/12h light cycle; water and food *ad libitum*). All animal experiments were approved by the Bezirksregierung Düsseldorf, Germany and performed in accordance with the Guide for the Care and Use of Laboratory Animals published by the NIH (NIH publication 85-23, revised 1996).

### Neutrophil Isolation and Polarization

Neutrophils were isolated from bone marrow from mice as described by Mocsai et al. (53) with some alterations. Mice were sacrificed by cervical dislocation. Bone marrow cells were flushed from the femurs and tibias with Ca^2+^-Mg^2+^-free HBSS with 20 mM HEPES and 0.5% FCS. Red blood cells were lysed using 0.2% NaCl, after which osmolarity was restored with 1.6% NaCl. The suspension was filtered through a 100-micron cell strainer to remove remaining bone parts and clots. After centrifugation, the pellet was dissolved in HBSS solution and layered on top a 62.5% Percoll layer. This was centrifuged 30 min at 1000 xg at RT without brakes, to separate the neutrophils from remaining bone marrow cells. After gradient centrifugation, the bottom layer, containing the neutrophils, was transferred to a separate tube and washed twice in HBSS solution. After this, cells were dissolved in medium (VLE Dulbecco DMEM with 3% FCS, 1 mM HEPES and 1% PenStrep) and 5-6 x 10^6^ cells per well were plated in a 6-well plate. Cells were left untreated or treated with 20 ng/mL IL-4, 10 ng/mL LPS and 2 ng/mL IFNγ, 10 ng/mL IGF1 or 10 or 100 ng/mL insulin for 4 hours at 37°C. In some experiments, additionally 250 or 5 nM of Janus kinase (Jak) family inhibitor (CAS 457081-03-7); 0.03 or 2.5 µM of Ruxolitinib; or 10 nm of BMS-911543 was added. After incubation, cells were collected, pelleted and resuspended in TRIzol or cell lysis buffer and stored at -20°C until further analysis.

### Real Time PCR and RNA Sequencing Transcript Expression Analysis

RNA isolation was performed using TRIzol reagent according to the manufacturer’s instructions. For real time PCR, 1 µg RNA was used to synthetize cDNA using the QuantiTect reverse transcription kit (Qiagen). qPCR was performed on the Step-One Plus real-time PCR system (Applied Biosystems) with Maxima SYBR Green and ROX qPCR Master Mix (Steinbrenner). Transcript quantities were calculated according to Sasse et al. (54) and normalized to NUDC mRNA. PCR primer sequences are given in **Table S3**.

For RNA sequencing, DNase digested total RNA samples used for transcriptome analyses were quantified (Qubit RNA HS Assay, Thermo Fisher Scientific) and quality measured by capillary electrophoresis using the Fragment Analyzer and the ‘Total RNA Standard Sensitivity Assay’ (Agilent Technologies, Inc. Santa Clara, USA). All samples in this study showed high quality RNA Quality Numbers (RQN; mean = 9.96). The library preparation was performed according to the manufacturer’s protocol using the ‘VAHTS™ Stranded mRNA-Seq Library Prep Kit for Illumina^®^’. Briefly, 300 ng total RNA were used for mRNA capturing, fragmentation, the synthesis of cDNA, adapter ligation and library amplification. Bead purified libraries were normalized and finally sequenced on the HiSeq 3000/4000 system (Illumina Inc. San Diego, USA) with a read setup of SR 1x150 bp. The bcl2fastq tool was used to convert the bcl files to fastq files as well for adapter trimming and demultiplexing.

Data analyses on fastq files were conducted with CLC Genomics Workbench (version 12.0.3, QIAGEN, Venlo. NL). The reads of all probes were adapter trimmed (Illumina TruSeq) and quality trimmed (using the default parameters: bases below Q13 were trimmed from the end of the reads, ambiguous nucleotides maximal 2). Mapping was done against the Mus musculus (mm10; GRCm38.86) (March 24, 2017) genome sequence. Statistical analysis was performed using Qlucore Omics explorer software. RPKM values were log2 transformed and prefiltered (threshold 0.1). After grouping of samples according to experimental conditions either multi-group comparison or two-group comparison functions were used. Samples with absolute fold change <1.5, and p<0.05 were considered to be differentially expressed genes (DGE). DGE were transferred to the Ingenuity Pathway Analysis (IPA) (Qiagen Inc. 2016) platform to identify possible upstream regulators which might have caused the transcriptional changes.

### Neutrophil Extracellular Trap Formation Assay

To induce NETosis, 150 000 neutrophils were seeded per well in a 96-well plate in 100 µl polarization medium without FCS. Neutrophils were then treated with the different polarizers IL-4 (20 ng/ml), IGF1 (10 ng/ml) in the presence or absence of 100 ng/ml PMA for 4 hours at 37°C. Then, 50 µl Quant-iT™ PicoGreen^®^dsDNA reagent (Thermo Fisher Scientific (P7581)) in a dilution of 1:100 was added to each well and incubated for 10 minutes at 37°C. After incubation, the fluorescence was measured using a fluorescence microplate reader and standard fluorescein wavelengths (excitation ~480 nm, emission ~520 nm).

### Phagocytosis Assay

The phagocytosis capacity of neutrophils was measured by flow cytometry using fluorescently labelled (FITC) Staphylococcus aureus (Thermo Fisher Scientific (S2851)). 150 000 neutrophils were seeded per well in 100 µl polarization medium in a 96-well plate. Neutrophils were then treated with the polarizer IL-4 (20 ng/ml) or IGF1 (10 ng/ml) for 4 hours at 37°C. Afterwards, neutrophils were incubated for 15 minutes with fluorescently labelled Staphylococcus aureus at a concentration of MOI 10. Thereafter, neutrophils are collected and washed with PBS. The fluorescence intensity of each cell was measured by flow cytometry, and presented as fold change compared to untreated.

### Western Blot

Cells were lysed in lysis buffer 4% SDS, 50mM Tris, 150mM NaCl, pH 7.4) supplemented with protease- and phosphatase inhibitors and were put in ultrasonic bath for 3 minutes for 3 times with 5 minutes break in between. Protein concentration was determined with a bicinchoninic acid assay (BSA) protein assay kit (Thermo Scientific). Equal amounts of protein were loaded on a 7.5 or 10% separating gel separated by SDS-PAGE and electrotransferred onto Protran nitrocellulose membranes in a PierceTM G2 Fast Blotter. Membranes were blocked in Odyssey blocking buffer (LI-COR Biosciences) and analyzed with antibodies against phospho-ERK (4307), AKT (2920), phospho-AKT Ser473 (9271), phospho-STAT1 (9167) from Cell Signaling Technology or phospho-STAT6 (700247) from Invitrogen. Secondary antibodies used were α-rabbit or α-mouse IRDye800CW and α-mouse IRDye680RD from LI-COR Biosciences. Signals were detected and quantified with an Odyssey near-infrared scanner (LI-COR Biosciences).

### Immunoprecipitation

Neutrophils used for immunoprecipitation were immediately after treatment dissolved in lysis buffer (10 mM Tris, 100 mM Natriumchlorid, 1% Igepal).

IGF1 receptor (#3027) and insulin receptor (#3025) antibodies from Cell Signaling Technology were bound to Protein A sepharose beads *via* the FC region of the IP antibody and subsequently incubated with the sample, resulting in formation of an antigen-antibody complex. This antigen-antibody complex was centrifuged in order to pellet the immune complex; the supernatant was removed. The beads were then washed and centrifuged three times to remove unspecific and unbound proteins. Finally, beads were western blotted as described above.

### Proximity Ligation Assay

Proximity Ligation Assay (Duolink^®^ *In Situ* Red Starter Kit Mouse/Rabbit, DUO92101, SigmaAldrich) was used according to manufacturers’ instruction to analyze interaction of the IGF1R and Jak2. In short, neutrophils were adhered to glass slides covered with Corning Cell tack in 24 well plates. They were left untreated, or treated 10 min with IGF1, after which they were fixed with 4% PFA and permeabilized with Triton (0.3%). Following washing steps, cells were blocked with Duolink blocking solution for 1 hour at 37°C. Subsequently, the primary antibody solution (IGF1 Receptor, AHO1292, Thermo Fisher Scientific and JAK1 (3344, Cell Signaling), JAK2 (3230, Cell Signaling), JAK3 (MA5-15561, Thermo Fisher) or TYK2 (PA5-119493, Thermo Fisher) was incubated over night at 4°C. Next day, cells were washed and incubated in PLA probe solution 1h at 37°C, after which cells were ligated 30 min at 37°C. Amplification was performed by adding polymerase for 100 min at 37°C. Cells were washed, nuclei were stained with DAPI and sealed using cover slips. Images were taken with a Zeiss LSM 880 Airyscan.

### *In Vivo* Myocardial Infarction

Myocardial infarction was induced in mice as described before (18, 55). In short, mice were anesthetized with 2% isoflurane, intubated and ventilated with oxygen-enriched gas (40% oxygen) using a Minivent microventilator (Hugo Sachs, Germany). To keep body temperature at 37.5°C, mice were placed in a supine position on a warming plate (Uno, Zevenaar, The Netherlands). For analgesia, the mice received buprenorphine (0.1 mg/kg body weight, subcutaneously (s.c.)). Electrocardiography (ECG) was recorded during the complete surgery. Thoracotomy was performed, the pericardium dissected and a 7-0 surgical prolene suture was passed underneath the LAD coronary artery 1 mm from the tip of the left atrium. Tightening the snare induced myocardial ischemia, which was confirmed by blanching of the myocardium and a ST-elevation in ECG. After 43 min ischemia, to mice obtained a bolus IGF1 (40 ng/g, Miltenyi Biotec, Bergisch Gladbach) or vehicle (0.1% BSA). Reperfusion was initiated after 45 min by opening the snare occluder, the suture was removed and the chest was closed. Micro-osmotic minipumps (Alzet, 1003D) were implanted s.c., to administer IGF1 (1 µg/g/day) or vehicle. After they gained spontaneous breathing, mice were extubated. After surgery, animals received buprenorphine (0.1 mg/kg s.c.) every 4 hours and in drinking water (0.009 mg/mL) over night for 3 days for analgesia.

### Echocardiography

Left ventricular function was analyzed before and 1 week after myocardial infarction using a Vevo2100 system (Visualsonics) equipped with a 30 MHz linear scanner as described previously (18, 55). Images were acquired at frame rates consistently above 200 frames/s. Mice were anesthetized with 2% isoflurane and placed in a supine position on a heated handling platform. ECG and breathing rates were monitored and body temperature was kept at 37°C with an infrared warming lamp, when required. The linear scanner was placed in a rail-based fixation system and Brightness (B)-mode movies of the parasternal long axis (PSLAX) and mid-ventricular, apical and basal orthogonal short axis were acquired by a blinded investigator. End diastolic (ED) and end systolic volumes (ESV) were determined by tracing of the endocardium in both diastole and systole. Simpson was used for analysis of ventricular volumes. Ejection fraction (EF) was calculated with the formula EF=((EDV-ESV)/EDV)*100.

### FACS Sorting

After 3 days of reperfusion, cardiac myeloid cells were isolated to perform single cell sequencing. To prevent blood contamination, hearts were retrograde perfused with PBS/heparin/Actinomycin D. A single-cell suspension was obtained *via* retrograde perfusion with digestion buffer (collagenase I (450 U/mL, Worthington Biochemical, LS004197), DNAse I (60 U/mL, Roche Diagnostics, 10104159001) and Actinomycin D in Hank’s balanced salt solution (HBSS) (Gibco, 14025)). After removal of atria, hearts were cut in ~1 mm³ pieces using a tissue chopper (McIlwain tissue chopper, Cavey Laboratory Engineering Co. Ltd.). Tissue was further digested for 30 min at 37°C in digestion buffer. Tissue clusters were triturated by pipetting 12 times using a 10 mL serological pipet after 10 and 20 min incubation, and 30 times using a 1 mL pipette at the end of digestion. After digestion, cells were filtered through a 100 µm filter and centrifuged 1 min at 50g to remove cardiomyocytes. Supernatant was filtered through a 40 µm filter, centrifuged at 300g for 10 min and the pellet was dissolved in FACS buffer (PBS with 0.5% BSA and 2 mM EDTA). Cells were incubated with Fc Block (Biolegend, 101302) for 10 min after which they were stained with CD45-FITC (Biolegend 103108) and CD11b- APC (BD Biosciences, 553312). To detect neutrophils in the single cell sequencing analysis, Ly6G TotalSeq antibody was added (Biolegend, 127659), and IGF1 and control animals were labeled with different Totalseq hashtag antibodies (Biolegend, 155831 and 155833) and incubated for 15 min. Cells were washed, propidium iodid was added to label dead cells, and living, single CD45^+^CD11b^+^ cells were FACS-sorted (MoFlo XDP, Beckman-Coulter). To reduce the influence of daily variations, on each experimental day one control and one IGF1 treated animal were prepared. Equal amounts of cells from each sample were combined after FACS-sorting. Cell quality was checked with trypan blue staining and visual inspection before starting single-cell RNA sequencing.

### Single Cell Library Generation

A total of ~16.000 cells were used as input for the single-cell droplet libraries generation for each sample (consisting of 1 control and 1 IGF1 sample) on the 10X Chromium Controller system utilizing the Chromium Single Cell 3’ NextGEM Reagent Kit v3.1 (10X Genomics, Pleasanton, CA, USA) according to manufacturer’s instructions. Sequencing was carried out on a NextSeq 550 system (Illumina Inc. San Diego, USA) with a mean sequencing depth of ~50.000 reads/cell.

### Processing of 10X Genomics Single Cell Data

Raw sequencing data was processed using the 10X Genomics CellRanger software (v3.1). Raw BCL-files were demultipexed and processed to Fastq-files using the CellRanger *mkfastq* pipeline. Alignment of reads to the mm10 genome and UMI counting was performed *via* the CellRanger *count* pipeline to generate a gene-barcode matrix. All samples were aggregated and normalized for sequencing depth using the cellranger *aggr* pipeline.

Further analyses were carried out with the Seurat v3.2 R package (56–58). Initial quality control consisted of removal of cells with less than fewer than 200 detected genes as well as removal of genes expressed in less than 3 cells. Furthermore, cells with a mapping rate of > 10% to the mitochondrial genome have been removed, as they represent dead or damaged cells. Demultiplexing based on cell labeling with hashtagging antibodies was also done in Seurat. Cell doublets have been removed from the dataset using DoubletFinder v2.0 (59). Normalization has been carried out utilizing SCTransform. Dimensional reduction of the data set was achieved by Principal Component analysis (PCA) based on identified variable genes and subsequent UMAP embedding. The number of meaningful Principal Components (PC) was selected by ranking them according to the percentage of variance explained by each PC, plotting them in an “Elbow Plot” and manually determining the number of PCs that represent the majority of variance in the data set. Cells were clustered using the graph-based clustering approach implemented in Seurat v3.0. Markers defining each cluster as well as differential gene expression between different clusters were calculated using a Wilcoxon Rank Sum test which is implemented in Seurat. After this initial analysis of all cells, we identified Neutrophils and Macrophages and performed a separate reclustering analysis with either of the two cells types using the workflow described above.

### Statistics

All data are presented as mean ± SD. Statistical analysis, with the exception of microarray and Sc-RNAseq data, was performed using Graph Pad Prism 7. qPCR data and Western blot data without inhibitors were analyzed by One-Way ANOVA, with Dunnett’s *post-hoc* test against untreated neutrophils. qPCR and Western blot data after inhibitor treatment were analyzed by One-Way ANOVA with Tukey’s *post-hoc* test. Results from the proximity ligation assay were analyzed using an independent T-test. For all statistical tests, a p<0.05 was considered significant.

## Data Availability Statement

The datasets presented in this study can be found in online repositories. The names of the repositories and accession numbers can be found below.

https://www.ncbi.nlm.nih.gov/geo/query/acc.cgi?acc=GSE198319

https://www.ebi.ac.uk/arrayexpress/experiments/E-MTAB-11590.

## Ethics Statement

The animal study was reviewed and approved by the Bezirksregierung Düsseldorf, Germany.

## Author Contributions

AG and RN conceived the study, designed the experiments, and supervised the project. RN, SR, AS, AH, and SG performed the experiments and analyzed the data. PP and KK performed the RNAseq analysis and TL and KK performed the ScRNAseq analysis. AG and RN wrote the manuscript. All authors critically reviewed and approved the manuscript.

## Funding

This work was funded by the German Research Foundation (CRC 1116 “Master Switches in Cardiac Ischemia”, TP A06 and the DFG program “Sequencing costs in projects”, SEQ2069) and by a grant of the Dr. Sigrid-Worch-Pöhler Stiftung and to AG and the Forschungskommission of the Heinrich Heine University to RN.

## Conflict of Interest

The authors declare that the research was conducted in the absence of any commercial or financial relationships that could be construed as a potential conflict of interest.

## Publisher’s Note

All claims expressed in this article are solely those of the authors and do not necessarily represent those of their affiliated organizations, or those of the publisher, the editors and the reviewers. Any product that may be evaluated in this article, or claim that may be made by its manufacturer, is not guaranteed or endorsed by the publisher.
