# Supplementary material for: Insulin-Like Growth Factor 1 Attenuates the Pro-Inflammatory Phenotype of Neutrophils in Myocardial Infarction
Source: Front Immunol. 2022 Jul 15;13:908023. doi: 10.3389/fimmu.2022.908023 (PMC9334797; doi:10.3389/fimmu.2022.908023)
Supplement: Supplementary file 1 [file Image_1.pdf]

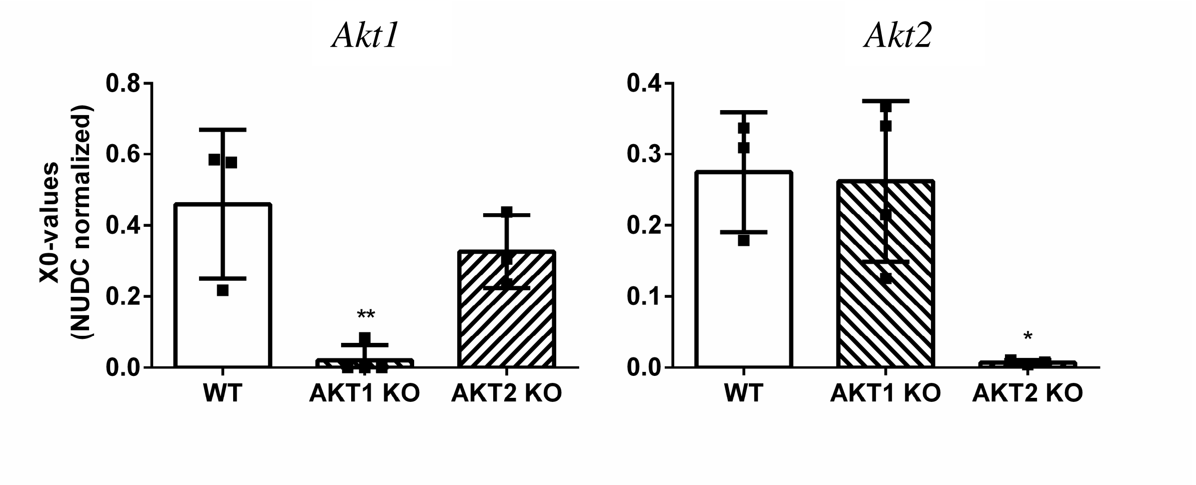

Supplementary Figure 1. RT-PCR of *Akt 1* and *Akt2* in Tie2-Cre Akt1$^{fl/fl}$ and Tie-2 Akt2-Cre Akt2$^{fl/fl}$ mice (related to Figure 3). *Akt1* and *2* expression in Tie2-Cre Akt1$^{fl/fl}$ and Tie2-Cre Akt2$^{fl/fl}$ mice was assessed by RT-PCR. X0 values normalized to *Nudc* (nudC nuclear distribution protein) expression of single measurements are shown. Bars represent mean ± SD. * $p<0.05$, ** $p<0.01$
