# Supplementary material for: Insulin-Like Growth Factor 1 Attenuates the Pro-Inflammatory Phenotype of Neutrophils in Myocardial Infarction
Source: Front Immunol. 2022 Jul 15;13:908023. doi: 10.3389/fimmu.2022.908023 (PMC9334797; doi:10.3389/fimmu.2022.908023)
Supplement: Supplementary file 2 [file Image_2.pdf]

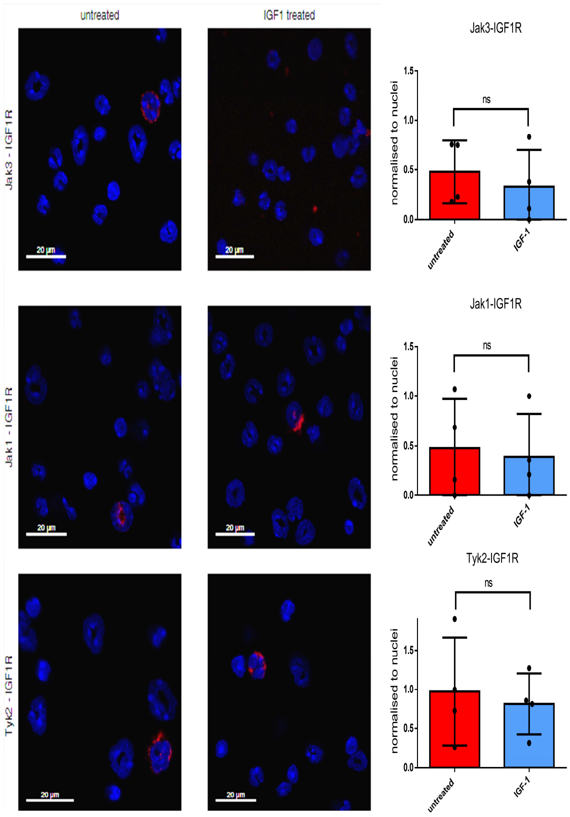

Supplementary Figure 2. Proximity ligation assay JAK1, JAK3 and TYK2 with the IGF1 receptor (Related to Figure 4). Representative images and analysis of untreated and IGF1 treated neutrophils after proximity ligation assay with JAK3, JAK1 and TYK2 and IGF1 receptor (IGF1R).
