# Supplementary material for: Insulin-Like Growth Factor 1 Attenuates the Pro-Inflammatory Phenotype of Neutrophils in Myocardial Infarction
Source: Front Immunol. 2022 Jul 15;13:908023. doi: 10.3389/fimmu.2022.908023 (PMC9334797; doi:10.3389/fimmu.2022.908023)
Supplement: Supplementary file 3 [file Image_3.pdf]

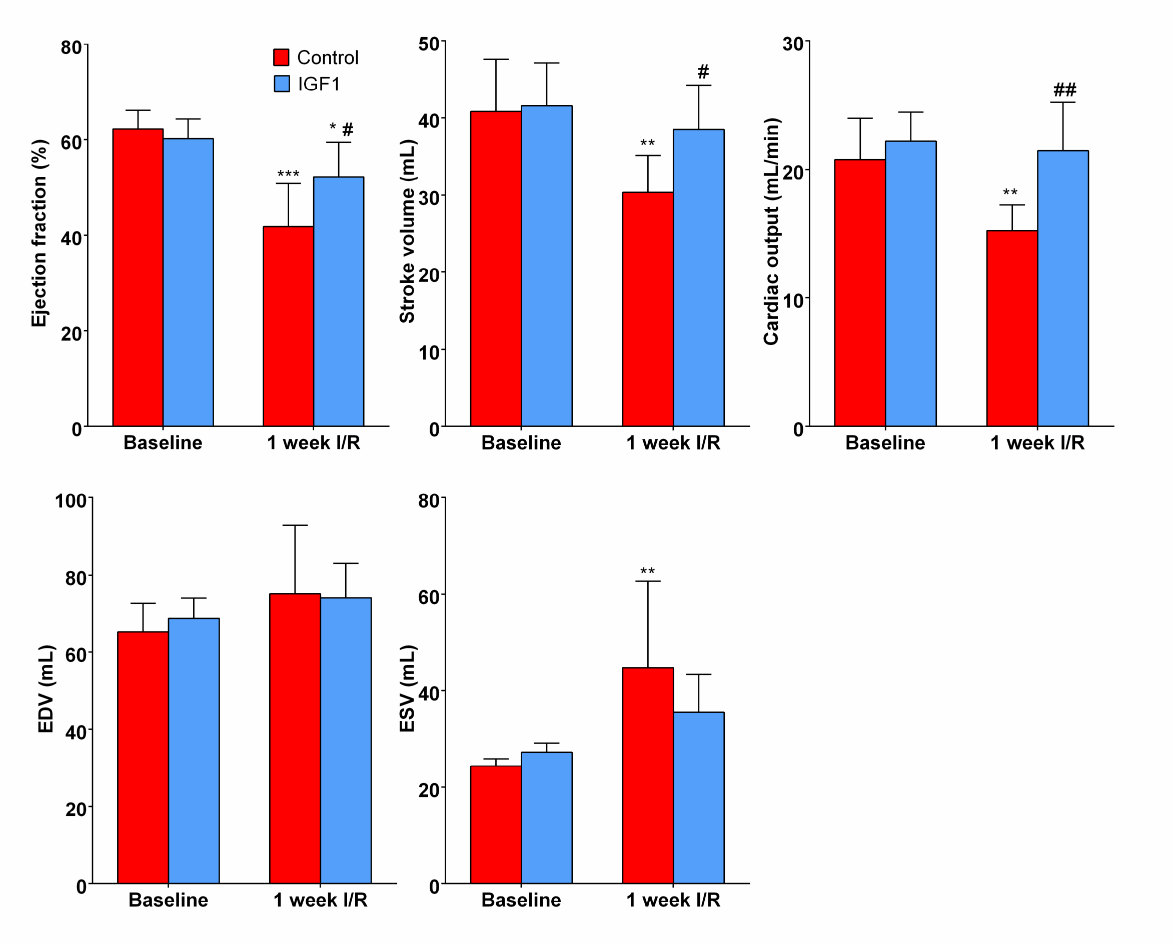

Supplementary Figure 3. IGF1 improves cardiac function after myocardial infarction (related to Figure 6). Ejection fraction, stroke volume, cardiac output, end-diastolic volume (EDV) and end-systolic volume (ESV) of mice at before and 1 week after myocardial infarction in control (red) and IGF1 (blue) treated mice. Bars represent mean ± SD. n=6 (control) or 8 (IGF1). * $p<0.05$, ** $p<0.01$, $p<0.001$ when compared to baseline. # $p<0.05$, ## $p<0.01$ when compared to control at the same time point.
