# Supplementary material for: Insulin-Like Growth Factor 1 Attenuates the Pro-Inflammatory Phenotype of Neutrophils in Myocardial Infarction
Source: Front Immunol. 2022 Jul 15;13:908023. doi: 10.3389/fimmu.2022.908023 (PMC9334797; doi:10.3389/fimmu.2022.908023)
Supplement: Supplementary file 4 [file Image_4.pdf]

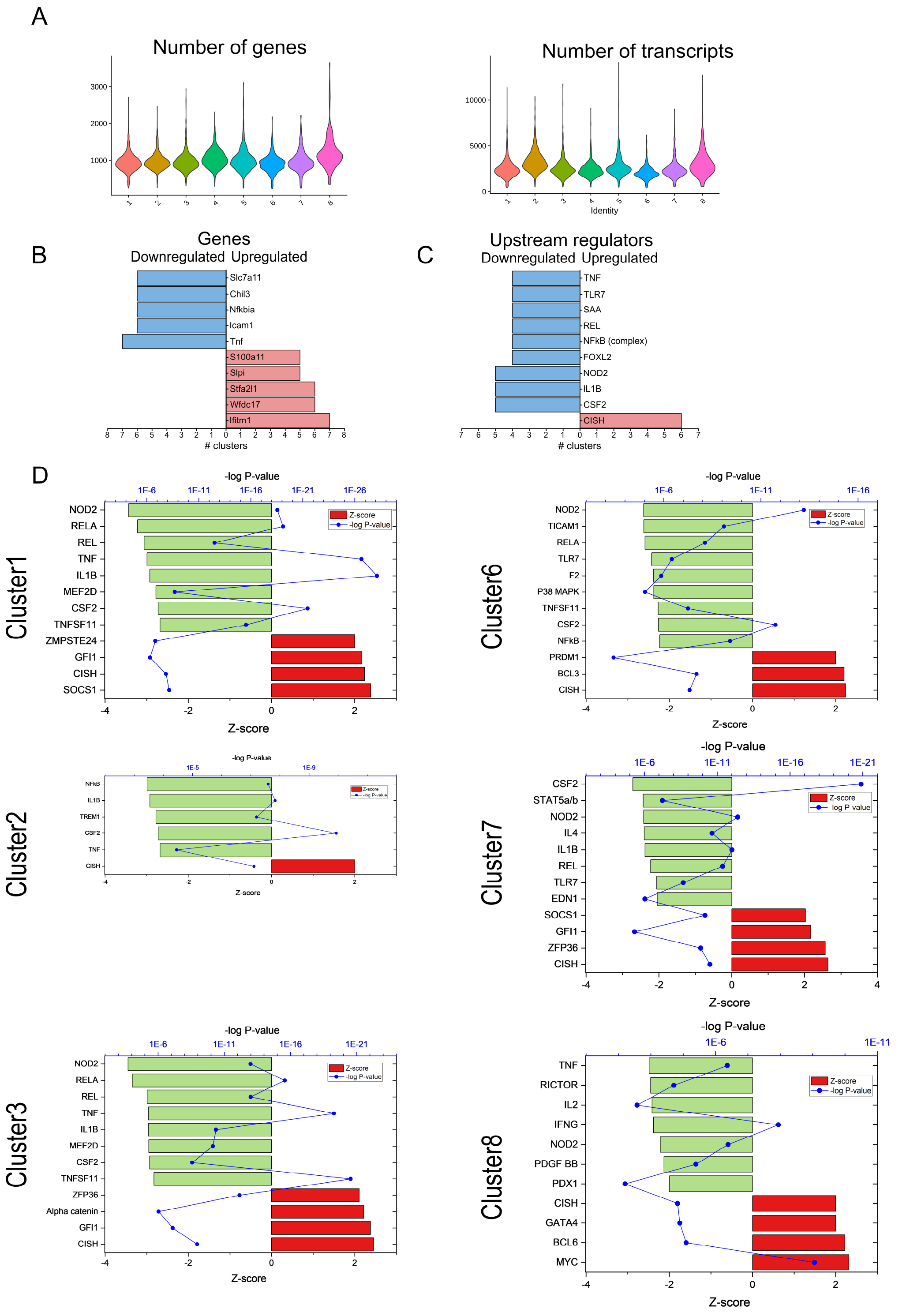

Supplementary Figure 4. IGF1 changes gene expression in neutrophils after MI (Related to Figure 6). (A) Violin plots of the number of transcript and number of genes within each neutrophil cluster. (B) The top 5 of up-and down regulated differentially expressed genes after IGF1 treatment and (C) The top 10 of upstream regulators changed after IGF1 treament. The x-axis shows to number of clusters in which the gene/upstream regulator is significantly up- (red, right) or downregulated (blue, left). (D) Upstream regulators that are affected after IGF1 treatment with a Z-score>2.0. The 12 most changed upstream regulators are shown.
