# Supplementary material for: Insulin-Like Growth Factor 1 Attenuates the Pro-Inflammatory Phenotype of Neutrophils in Myocardial Infarction
Source: Front Immunol. 2022 Jul 15;13:908023. doi: 10.3389/fimmu.2022.908023 (PMC9334797; doi:10.3389/fimmu.2022.908023)
Supplement: Supplementary file 5 [file Image_5.pdf]

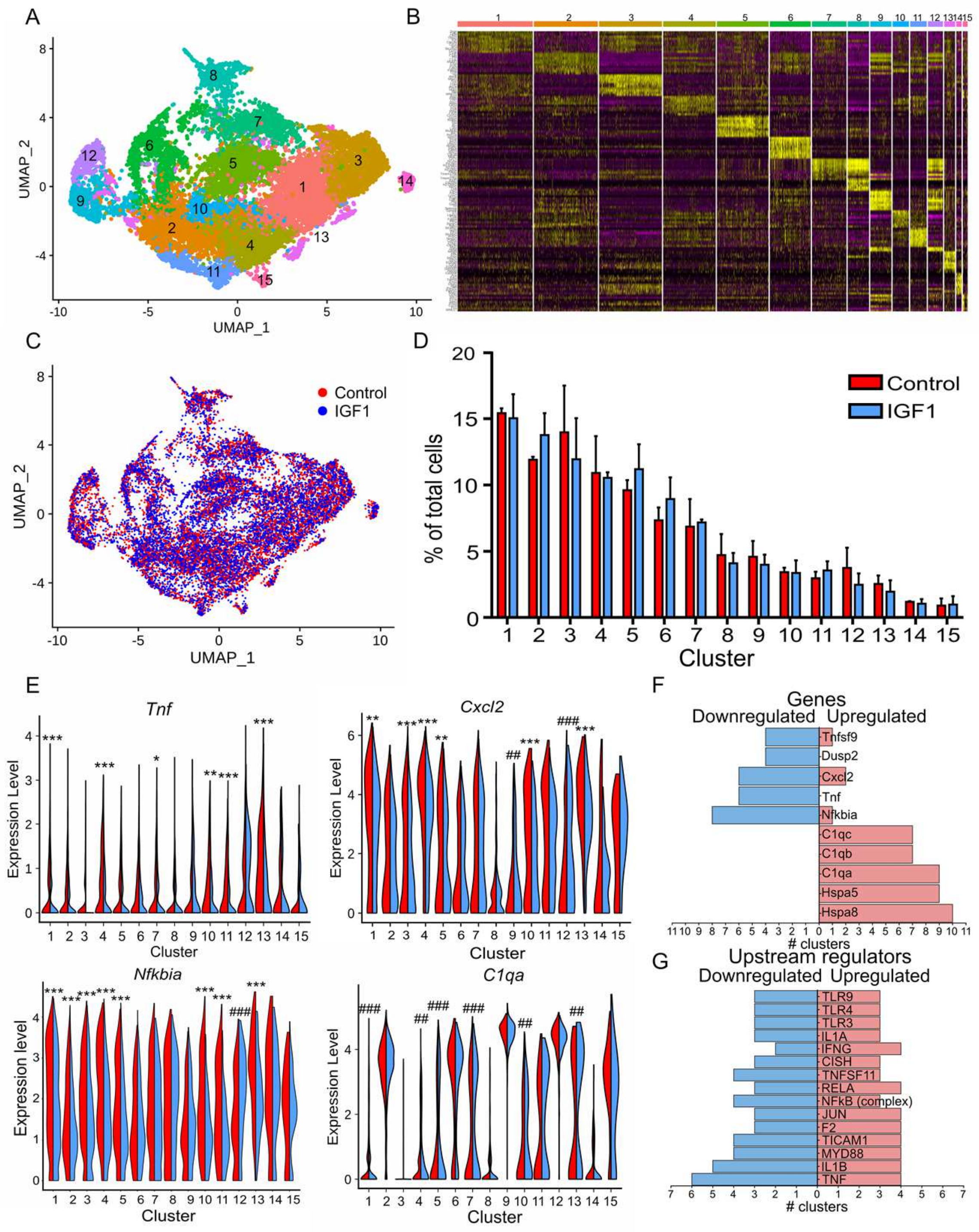

Supplementary Figure 5. IGF1 treatment creates an anti-inflammatory phenotype of macrophages after myocardial infarction (related to Figure 6). (A) Reclustering of non-neutrophil myeloid cells with a higher resolution resulted in 15 different clusters. (B) Heatmap depicting the top 10 differently expressed genes for each cluster. (C) UMAP showing control (red) and IGF1 treated (blue) non-neutrophil myeloid cells does not show a clear differences in the distribution of cells over the clusters. (D) The amount of cells per cluster as percentage of the total number of non-neutrophil myeloid cells. (E) Violin plots show a clear difference in the expression of *Tnf*, *Nfkbia, Cxcl2* and *C1qa* in multiple clusters between control (red, left) and IGF1 (blue, right) treated cells. (F) The top 5 of up-and down regulated differentially expressed genes after IGF1 treatment. The x-axis shows to number of clusters in which the gene is significantly up- (red, right) or downregulated (blue, left). (G) Top 10 upstream regulators affected after IGF1 treatment as determined by IPA analysis. * $p<0.05$ higher in control, ** $p<0.01$ higher in control *** $p< 0.001$ higher in control, ## $p<0.01$ higher in IGF1, ### $p<0.001$ higher in IGF1
