# Supplementary material for: Insulin-Like Growth Factor 1 Attenuates the Pro-Inflammatory Phenotype of Neutrophils in Myocardial Infarction
Source: Front Immunol. 2022 Jul 15;13:908023. doi: 10.3389/fimmu.2022.908023 (PMC9334797; doi:10.3389/fimmu.2022.908023)
Supplement: Supplementary file 8 [file Table_3.docx]

Table S3. PCR primer

| Name | Direction | Sequence 5’-3’ | Manufacturer |
| --- | --- | --- | --- |
| *Nudc* | Fwd  Rev | AGAACTCCAAGCTATCC  CTTCAGGATTTCCTGTTC | Sigma-Aldrich, USA |
| *Tnf* | Fwd  Rev | CTATGTCTCAGCCTCTTCTC CATTTGGGAACTTCTCATCC | Sigma-Aldrich, USA |
| *Nos*2 | Fwd  Rev | CATCACCAGTATTATGGCTC  TTTCCTTTGTTACAGCTTCC | Sigma-Aldrich, USA |
| *Il12a* | Fwd  Rev | GAAGACATCATGAAGAC  CTCTTGTTGTGGAAGAAGTC | Sigma-Aldrich, USA |
| *Arg1* | Fwd  Rev | CTGACCTATGTGTCATTTGG  CATCTGGGAACTTTCCTTTC | Sigma-Aldrich, USA |
| *Retnla* | Fwd  Rev | GATGAAGACTACAACTTGTTCC  AGGGATAGTTAGCTGGATTG | Sigma-Aldrich, USA |
| *Chi3l3* | Fwd  Rev | TCACAGGTCTGGCAATTCTTCTG  TTTGTCCTTAGGAGGGCTTCCTCG | Invitrogen, USA |
| *Car4* | Fwd  Rev | GCATTTATGATTGAGGTAGGAG  AATGGGGTTTGGAGATACTG | Sigma-Aldrich, USA |
| *Slc28a3* | Fwd  Rev | CATTTAAGATCCTGCCCATC  CCAATAAATATGTTGCCAGC | Sigma-Aldrich, USA |
| *Fam19a3* | Fwd  Rev | AAAACCAGACAACACCTAAG  CCATCTGATGAACAATATGGC | Sigma-Aldrich, USA |
